# Localization of Double-Strand Break Repair Proteins to Viral Replication Compartments following Lytic Reactivation of Kaposi's Sarcoma-Associated Herpesvirus

**DOI:** 10.1128/JVI.00930-17

**Published:** 2017-10-27

**Authors:** Robert Hollingworth, Richard D. Horniblow, Calum Forrest, Grant S. Stewart, Roger J. Grand

**Affiliations:** Institute of Cancer and Genomic Sciences, University of Birmingham, Birmingham, United Kingdom; University of Southern California

**Keywords:** ATM, DDR, DNA repair, DNA-PK, herpesvirus, Kaposi's sarcoma-associated herpesvirus, lytic replication, MRN complex, NHEJ, replication compartments

## Abstract

Double-strand breaks (DSBs) in DNA are recognized by the Ku70/80 heterodimer and the MRE11-RAD50-NBS1 (MRN) complex and result in activation of the DNA-PK and ATM kinases, which play key roles in regulating the cellular DNA damage response (DDR). DNA tumor viruses such as Kaposi's sarcoma-associated herpesvirus (KSHV) are known to interact extensively with the DDR during the course of their replicative cycles. Here we show that during lytic amplification of KSHV DNA, the Ku70/80 heterodimer and the MRN complex consistently colocalize with viral genomes in replication compartments (RCs), whereas other DSB repair proteins form foci outside RCs. Depletion of MRE11 and abrogation of its exonuclease activity negatively impact viral replication, while in contrast, knockdown of Ku80 and inhibition of the DNA-PK enzyme, which are involved in nonhomologous end joining (NHEJ) repair, enhance amplification of viral DNA. Although the recruitment of DSB-sensing proteins to KSHV RCs is a consistent occurrence across multiple cell types, activation of the ATM-CHK2 pathway during viral replication is a cell line-specific event, indicating that recognition of viral DNA by the DDR does not necessarily result in activation of downstream signaling pathways. We have also observed that newly replicated viral DNA is not associated with cellular histones. Since the presence and modification of these DNA-packaging proteins provide a scaffold for docking of multiple DNA repair factors, the absence of histone deposition may allow the virus to evade localization of DSB repair proteins that would otherwise have a detrimental effect on viral replication.

**IMPORTANCE** Tumor viruses are known to interact with machinery responsible for detection and repair of double-strand breaks (DSBs) in DNA, although detail concerning how Kaposi's sarcoma-associated herpesvirus (KSHV) modulates these cellular pathways during its lytic replication phase was previously lacking. By undertaking a comprehensive assessment of the localization of DSB repair proteins during KSHV replication, we have determined that a DNA damage response (DDR) is directed to viral genomes but is distinct from the response to cellular DNA damage. We also demonstrate that although recruitment of the MRE11-RAD50-NBS1 (MRN) DSB-sensing complex to viral genomes and activation of the ATM kinase can promote KSHV replication, proteins involved in nonhomologous end joining (NHEJ) repair restrict amplification of viral DNA. Overall, this study extends our understanding of the virus-host interactions that occur during lytic replication of KSHV and provides a deeper insight into how the DDR is manipulated during viral infection.

## INTRODUCTION

Kaposi's sarcoma-associated herpesvirus (KSHV) is a human tumor virus that is responsible for the vascular malignancy known as Kaposi's sarcoma (KS) as well as the B cell lymphoproliferative disorders primary effusion lymphoma (PEL) and multicentric Castleman's disease (MCD) (1–3). KSHV typically establishes an asymptomatic lifelong latent infection characterized by a restricted viral gene expression program that facilitates evasion of host immune surveillance. However, in order to amplify its genetic material and produce infectious virions, KSHV utilizes an alternate life cycle phase known as lytic replication. Lytic replication of herpesviruses involves regulated and sequential expression of viral genes that are divided into classes known as immediate early, early, and late (4). KSHV immediate early viral genes include ORF50/RTA, which is required for initiation of the full lytic cascade and transactivates other viral genes required for complete lytic replication (5, 6). Early lytic gene products include six viral replication factors required for synthesis of viral DNA as well as other proteins that modulate host cell signaling pathways (7). Late viral genes, expression of which occurs following the onset of viral genome amplification, typically encode structural proteins that make up the mature viral capsid (8). Lytic replication of herpesviruses involves the formation of large globular domains in the host cell nucleus known as viral replication compartments (RCs), which contain newly synthesized viral DNA. The precise nature and function of these viral RCs remain under investigation, although it has been suggested that they serve to concentrate viral and cellular proteins required for viral replication while at the same time shielding viral genomes from detection by the cellular immune machinery (9). Both theta-type and rolling-circle replication mechanisms are believed to play a role in lytic replication of herpesvirus DNA, the end products of which are long concatemers containing unit-length viral genomes (10). Recombination is also known to occur frequently between viral genomes, while a recombination-dependent mechanism could also contribute to the generation of concatemeric viral DNA (11).

The DNA damage response (DDR) encompasses multiple signaling pathways responsible for the detection and reversal of DNA lesions that can result from the actions of endogenous cellular processes and exogenous agents. Proteins that participate in this response are often categorized as either sensor, transducer, or effector molecules, depending on their role within DDR signaling cascades. DNA lesions can take many forms, although double-strand breaks (DSBs), in which both strands of the double helix are severed, are particularly cytotoxic due to their tendency to cause translocations, fusions, and deletions when incorrectly repaired (12). ATM and DNA-PK are two members of the phosphatidylinositol 3-kinase-related kinase family of enzymes that regulate the response to cellular DSBs (13). The MRE11-RAD50-NBS1 (MRN) complex, which is involved in DNA replication, DNA repair, and checkpoint activation, is often described as a “first responder” to DNA damage, as it recognizes and binds DSBs and plays a role in ATM recruitment and activation (14). ATM is rapidly activated following the formation of DSBs and orchestrates the cellular response to this DNA lesion by phosphorylating multiple substrates that regulate cell cycle checkpoints, DNA repair, and apoptosis (15). Both ATM and DNA-PK can phosphorylate the histone variant H2AX on serine 139 (γH2AX) on either side of the break site, which allows binding of MDC1 to H2AX via its BRCT repeat domain (16–18). Subsequent phosphorylation of MDC1 by ATM allows recruitment of the E3 ubiquitin ligase RNF168, which ubiquitylates histone H2A on K15 to allow binding of 53BP1 (19–21). 53BP1 plays a role in DNA repair and checkpoint activation and is used as a specific marker of DSBs in immunofluorescence (IF) microscopy (22). Another key substrate of ATM is CHK2, activation of which can promote cell cycle arrest and apoptosis through phosphorylation of multiple proteins, including p53 and CDC25A (23, 24).

DNA-PK is a central component of the nonhomologous end joining (NHEJ) repair pathway, which operates throughout the cell cycle and involves direct religation of severed DNA molecules. During NHEJ, the Ku70/80 heterodimer recognizes and binds DSBs before recruiting the DNA-PK catalytic subunit (DNA-PKcs) to form the active DNA-PK holoenzyme. Numerous factors are involved in DNA end processing before the DNA ligase IV/XRCC4 complex ligates the break, facilitated by accessory proteins such as XLF and PAXX (25, 26). The other principal pathway that corrects DSBs is known as homologous recombination (HR) and is active only in the S and G_2_ phases when a sister chromatid is available to provide a template for accurate repair. HR is preceded by localized DNA resection that is initiated by the MRN complex and is enhanced by recruitment of BRCA1 and CtIP (27, 28). The trimeric RPA complex protects the resulting single-stranded DNA (ssDNA) before BRCA2 or RAD52 mediates its replacement by RAD51 to form a nucleofilament that invades the undamaged homologous DNA strand (29). Following homology searching and annealing of the nucleofilament to a homologous undamaged region, DNA polymerases extend the damaged strand, while nucleases then resolve the resulting DNA structures to complete the repair process (30).

It is now clear that herpesviruses interact extensively with pathways responsible for detection and repair of cellular DSBs, and these interactions occur at multiple stages of their reproductive cycles. DSB repair proteins such as DNA-PKcs and BRCA1, for example, can interact with herpesvirus genomes following *de novo* infection and trigger innate immune responses against the virus (31, 32). Activation of the ATM-CHK2 arm of the DDR has also been observed during replication of several species of herpesvirus, including KSHV, Epstein-Barr virus (EBV), herpes simplex virus 1 (HSV-1), human cytomegalovirus (HMCV), and murine gammaherpesvirus 68 (MHV-68), although there has been disagreement regarding the contribution of this event to successful viral replication (33–37). While DSB repair proteins participate in the cellular antiviral response, they have also been observed at sites of herpesvirus DNA synthesis in the nucleus, where they have been implicated in efficient replication of viral genomes (38, 39). Factors that participate in HR repair, such as RPA32, MRE11, RAD51, and RAD52, for example, have been shown to directly associate with replicating EBV DNA, while knockdown of RPA32 and RAD51 dramatically attenuates EBV lytic replication (38).

In the case of KSHV, activation of a DSB-specific DDR has been demonstrated following initial viral entry and during the lytic replication program, and in both cases some aspects of DDR activation appear to play a positive role in the viral life cycle (33, 40). The KSHV lytic protein ORF57/MTA has also been shown to cause DSBs when expressed alone through the formation of R loops in cellular DNA (41). We have previously reported that KSHV lytic replication in B cells leads to activation of a DSB-specific DDR that includes activation of the ATM and DNA-PK kinases and phosphorylation of their downstream substrates (33). Inhibition of viral DNA amplification and late lytic gene expression did not reduce phosphorylation of DDR proteins, indicating that immediate early and early lytic gene expression are sufficient to cause DDR activation.

In the current study, we have undertaken a comprehensive assessment of the localization of DSB repair proteins in cells containing lytic KSHV and have also evaluated the contribution of key regulators of the DSB response to viral replication efficiency. Using IF microscopy, we revealed that during the KSHV lytic replication program, protein complexes involved in the initial recognition of DSBs consistently colocalize with viral genomes within RCs while DSB repair foci also form outside viral replication domains. We also demonstrate that cellular histones are not associated with newly amplified viral DNA and that recognition of viral genomes by the DSB-sensing apparatus does not necessarily activate wider DDR signaling cascades. Overall, these results highlight significant interplay between KSHV and the cellular DSB repair machinery following viral reactivation and reveal both positive and negative consequences of these interactions for the viral life cycle.

## RESULTS

### DSB-sensing proteins are recruited to KSHV RCs.

As outlined above, DSB repair pathways are initiated following recognition of DNA lesions by sensor proteins. To determine if proteins involved in initial detection of DSBs localize to KSHV genomes during the lytic cycle, IF microscopy was carried out on EA.hy926-RTA cells containing lytic KSHV. KSHV RCs were identified using antibodies against the viral ssDNA binding protein (SSB) or the viral processivity factor (PF8), which are both involved in the replication of KSHV DNA. For each DSB repair protein assessed, a minimum of 100 cells containing viral RCs were examined for each of three independent experiments.

**FIG 1:**
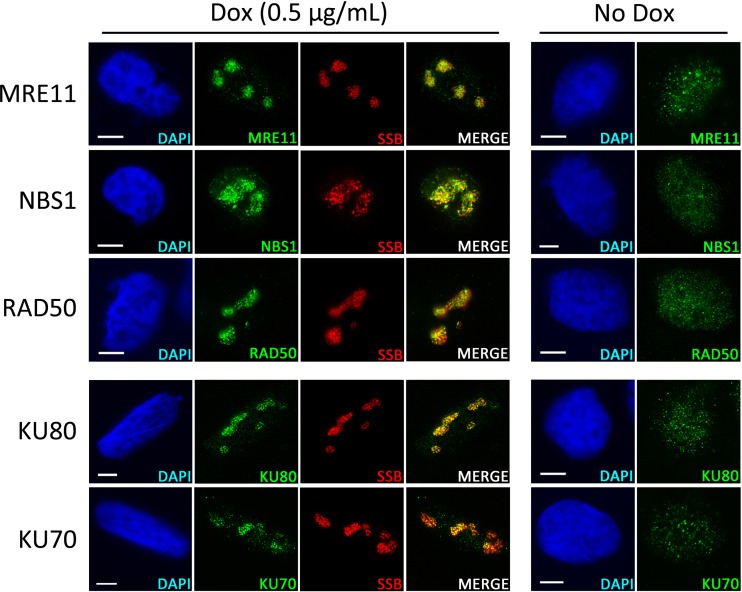
Localization of DSB-sensing complexes during lytic replication of KSHV. EA.hy926-RTA cells containing latent KSHV were treated with doxycycline (Dox) for 48 h or left untreated prior to IF analysis of nuclear localization of MRE11, NBS1, RAD50, Ku70, and Ku80 in relation to KSHV RCs marked by the viral SSB protein. Scale bars represent 5 μm.

Using antibodies against MRE11, NBS1, and RAD50, we observed that the complete MRN complex, which recognizes and binds DSBs, is present at KSHV RCs following viral reactivation (Fig. 1). The MRN complex was observed tightly associated with RCs in all of the cells examined and was found to localize to these replication domains as soon as they were detectable, and it also remained associated as RCs expanded to fill the nucleus (data not shown).

Another protein complex that associates with DSBs is the Ku70/80 heterodimer, which precedes recruitment of DNA-PKcs to form the DNA-PK holoenzyme that is required for NHEJ repair (42). As with the MRN complex, both Ku70 and Ku80 were observed localized to KSHV RCs in all of the cells examined (Fig. 1). For all the DNA repair proteins assessed using IF microscopy, staining was carried out using a preextraction step which removes the majority of unbound nucleoplasmic protein but preserves the chromatin-associated fraction (43). The presence of both the MRN complex and Ku70/80 at KSHV RCs following preextraction suggests that these proteins were associating with replicating viral genomes. Overall, these images indicate that amplification of the KSHV genome involves the formation of DSBs in viral DNA that are recognized by the cellular DDR.

### Downstream HR proteins are not consistently observed at KSHV RCs.

**FIG 2:**
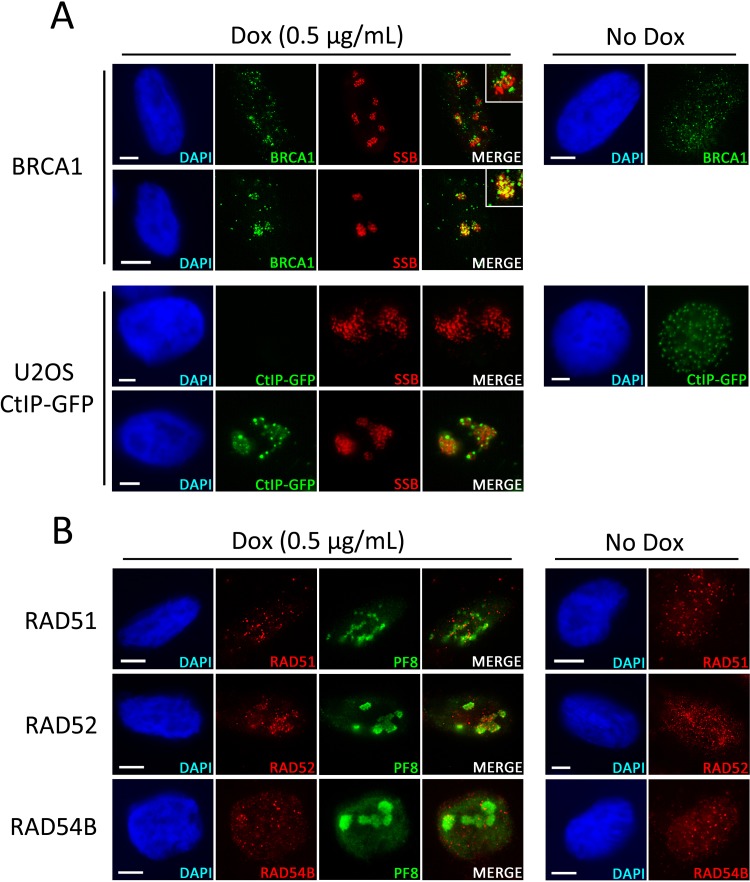
Localization of HR repair factors during lytic replication of KSHV. (A) EA.hy926-RTA cells containing latent KSHV were treated with doxycycline for 48 h or left untreated prior to IF analysis of nuclear localization of BRCA1 in relation to KSHV RCs marked by the viral SSB protein. Latently infected U2OS cells expressing GFP-CtIP were treated with sodium butyrate for 48 h or left untreated prior to IF analysis of GFP-CtIP localization in relation to KSHV RCs marked by the viral SSB protein. (B) EA.hy926-RTA cells were prepared as described above prior to IF analysis of nuclear localization of the HR repair factors RAD51, RAD52, and RAD54B in relation to KSHV RCs marked by the viral PF8 protein. Scale bars represent 5 μm.

Following localization to DSBs, the MRN complex is important for the early stages of HR repair by initiating DNA resection (44). The efficiency of DNA resection has been found to be enhanced by the formation of a complex involving MRN, BRCA1, and CtIP (27). We therefore examined the localization of BRCA1 and CtIP during KSHV lytic replication to determine whether these factors were also associated with viral RCs (Fig. 2A). As before, the localization of these proteins was examined in a minimum of 100 cells containing KSHV RCs in each of three independent experiments. In contrast to members of the MRN complex, BRCA1 was not seen at KSHV RCs in the majority of the cells examined. However, in 22% of the cells assessed, at least some BRCA1 protein could be observed associated with KSHV genomes, while in those cells where BRCA1 did not localize to viral genomes, the protein often formed visible foci in the vicinity of viral RCs (Fig. 2A). Due to the lack of a commercial CtIP antibody that is compatible with IF microscopy, the localization of this protein was assessed by infection of a previously developed U2OS cell line that expresses doxycycline-inducible green fluorescent protein (GFP)-tagged CtIP (45) (Fig. 2A). Similar to BRCA1, CtIP was typically not observed tightly associated with viral genomes and was often not detectable in cells containing lytic virus. However, in 15% of the cells examined, GFP-CtIP was observed localized to viral RCs. These results suggest that despite the consistent localization of the MRN complex to viral genomes, DSB repair factors that associate with MRN are only occasionally recruited to KSHV replication domains.

Additional factors involved in HR repair downstream of the DNA resection stage have previously been implicated in replication of herpesvirus DNA (38, 46). We therefore examined the localization of several other proteins known to participate in HR: RAD51, which forms nucleoprotein filaments that invade homologous DNA templates; RAD52, which promotes RAD51 loading and cDNA annealing; and RAD54B, which is involved in localized chromatin remodelling (47–49) (Fig. 2B). In contrast to the consistent localization of the MRN complex and Ku70/80, RAD51 and RAD54B were never observed localized to KSHV RCs in any of the cells examined. However, in 82% of the cells assessed, RAD52 was observed concentrated in and around sites of viral replication, although it did not appear to be as tightly associated with these replication domains as MRN and Ku70/80. It appears, therefore, that while DSB-sensing complexes are always observed at viral RCs, downstream HR repair proteins are not consistently recruited to these viral replication sites.

### DSB repair foci form outside KSHV RCs.

**FIG 3:**
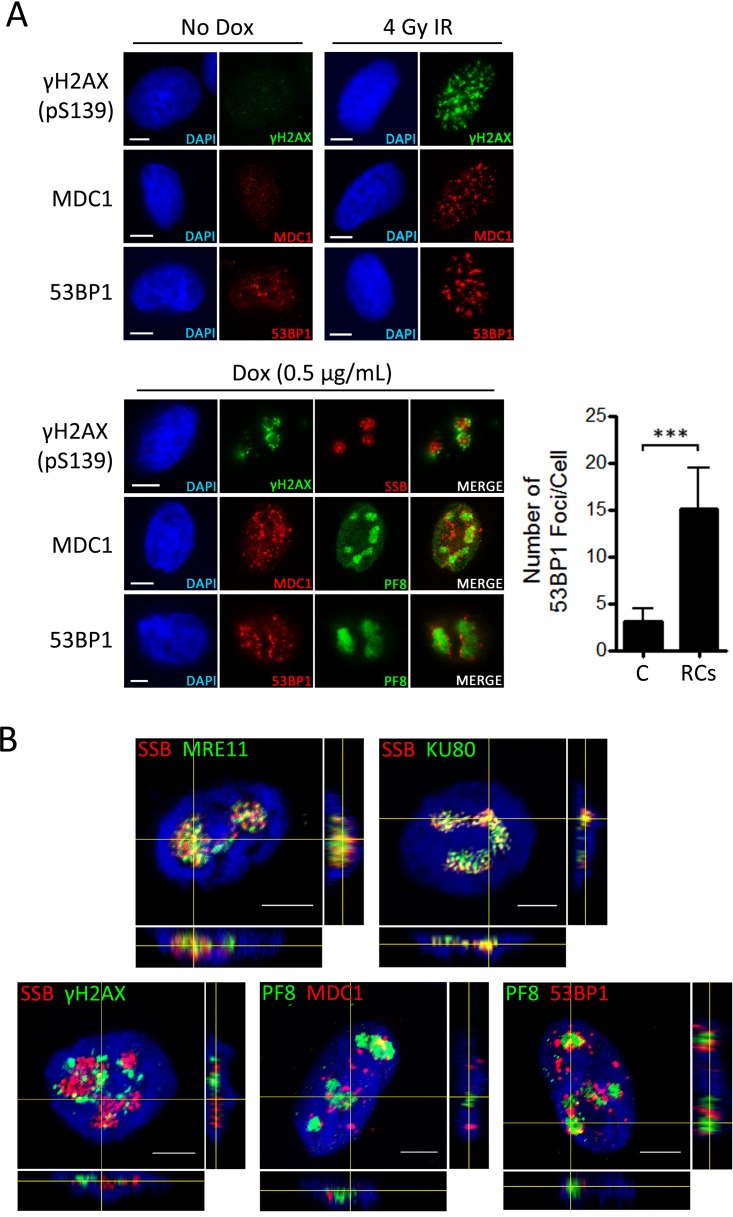
Formation of DSB repair foci during lytic replication of KSHV. (A) EA.hy926-RTA cells were exposed to 4 Gy IR for 1 h or left untreated prior to IF analysis of γH2AX, MCD1, and 53BP1 focus formation. EA.hy926-RTA cells containing latent KSHV were then treated with doxycycline for 48 h to induce lytic replication prior to IF analysis of the same DDR proteins in relation to KSHV RCs marked by the viral SSB or PF8 protein. The numbers of 53BP1 foci in cells containing KSHV RCs (RCs) and in cells that do not contain lytic virus (C) were counted in at least 50 cells per condition in three independent experiments. All data bars represent the mean from three independent experiments, while error bars signify the standard error of the mean (SEM). ***, *P* < 0.001 (statistical analyses were performed using a two-tailed and unpaired Student *t* test). (B) Confocal scanning microscopy was used to generate composite micrographs from z-stack images of EA.hy926-RTA cells containing lytic KSHV. The images demonstrate localization of MRE11, Ku80, γH2AX, MDC1, and 53BP1 in relation to KSHV RCs marked by the viral SSB or PF8 proteins. The side panels provide orthogonal views (*xz* and *yz*), while crosshairs show the cut point through the *x*, *y*, and *z* axes of the image. Scale bars represent 5 μm.

Since the presence of Ku70/80 and the MRN complex at sites of KSHV replication suggests the formation of DSBs in viral DNA, the localization of other proteins that form foci in response to DSBs was also examined in relation to KSHV RCs (Fig. 3). Phosphorylation of H2AX at either side of DSBs facilitates an interaction between γH2AX and MDC1 that subsequently leads to recruitment of 53BP1 (17, 19). IF staining was therefore carried out using antibodies against γH2AX, MDC1, and 53BP1 in control EA.hy926-RTA cells and in EA.hy926-RTA cells treated with 4 Gy ionizing radiation (IR) to demonstrate the typical distribution of these proteins in both unperturbed cells and cells containing high levels of DNA damage (Fig. 3A). In the absence of lytic replication or IR exposure, γH2AX and MDC1 both displayed a faint and diffuse nuclear staining pattern, while 53BP1 was either diffuse or concentrated in several foci that typically numbered less than five per cell. In contrast, following exposure to IR, all three proteins formed numerous discrete foci across the nucleus, indicating the occurrence of cellular DSBs. Staining for these proteins was then carried out in EA.hy926-RTA cells containing lytic KSHV (Fig. 3A). All three DSB repair factors formed foci in cells containing viral RCs, indicating that lytic replication leads to formation of DSBs. However, unlike Ku70/80 and members of the MRN complex, these factors were typically observed outside viral RCs and were often concentrated at the margins of viral replication domains. Since 53BP1 foci are often used as a specific marker of DSBs, the number of 53BP1 foci in cells containing KSHV RCs was then quantified in at least 50 cells in three independent experiments and compared with the number of foci in uninfected cells (Fig. 3A). This analysis revealed a significant increase in the number of 53BP1 foci in cells containing lytic virus, indicating an increase in the formation of DSBs during this stage of the viral life cycle.

To confirm the localization of MRE11, Ku80, γH2AX, MDC1, and 53BP1 in relation to KSHV RCs, confocal microscopy was used to generate composite micrographs with orthogonal views from stacked images of EA.hy926-RTA cells containing viral replication domains (Fig. 3B). These images confirm that MRE11 and Ku80 colocalize with viral genomes within RCs, while γH2AX, MDC1, and 53BP1 form foci that are typically concentrated on the periphery of viral RCs. These images confirm that while DSB sensors localize to viral genomes, DSB repair foci also form outside viral RCs, suggesting that DNA lesions occur in cellular chromatin during the lytic replication program.

### Cellular histones do not associate with amplified viral DNA.

**FIG 4:**
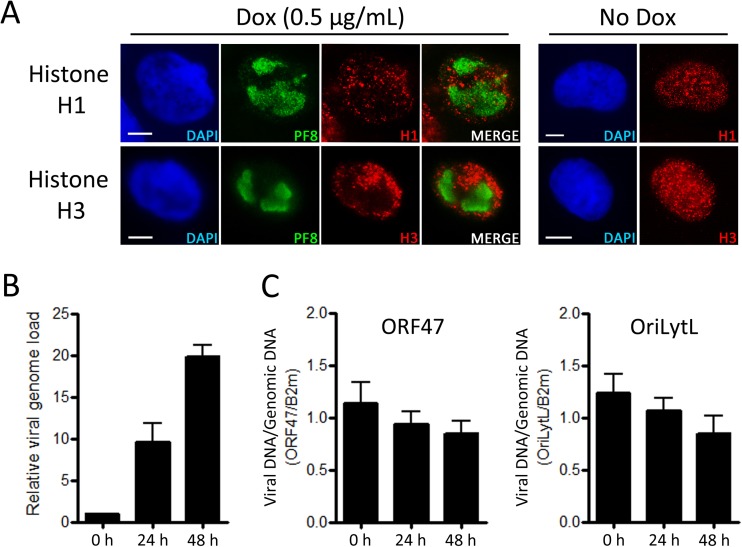
Association of cellular histones with amplified viral genomes. (A) EA.hy926-RTA cells containing latent KSHV were treated with doxycycline for 48 h or left untreated prior to IF analysis of the distribution of histones H1 and H3 in relation to KSHV RCs marked by the viral PF8 protein. Scale bars represent 5 μm. (B and C) TREx BCBL-1-RTA cells were treated with doxycycline for 0, 24, and 48 h and chromatin preparations were prepared by sonication. (B) Following DNA cleanup, the viral genome load was determined using qPCR relative to the 0-h control. (C) Chromatin preparations were subjected to ChIP for histone H3 or control IgG. Viral DNA was detected in immunoprecipitated material by qPCR using primers specific to the ORF47 or OriLytL region of the KSHV genome. Following subtraction of background readings, values were normalized to cellular DNA detected using primers specific to β2-microglobulin (Bm2). All data bars represent the mean from three independent experiments, while error bars signify the SEM.

While Ku70/80 and the MRN complex have an affinity for double-stranded DNA ends, recruitment of the DSB repair factors MDC1 and 53PB1 and the formation of γH2AX foci at DSB sites require the presence and modification of cellular histones (18, 19, 50). While latent KSHV genomes are known to associate with histones, it is not clear whether KSHV DNA synthesized during the lytic phase also associates with these cellular proteins. To determine if histones associate with newly amplified KSHV DNA, we first examined the distribution of histones H1 and H3 in relation to KSHV RCs marked by PF8 (Fig. 4A). This initial IF analysis indicated that these cellular histones are not concentrated within KSHV RCs and are therefore not likely to be associated with replicating viral DNA.

We then used chromatin immunoprecipitation (ChIP) to determine if histone H3 is enriched on viral DNA during amplification of KSHV genomes in B cells. TREx BCBL-1-RTA cells were treated with doxycycline for 0, 24, and 48 h, and chromatin preparations were prepared by sonication. When these input chromatin preparations were cleaned up and subjected to quantitative PCR (qPCR) analysis, there was an expected increase in the amounts of viral DNA at 24 and 48 h following doxycycline treatment relative to that for the 0-h controls (Fig. 4B). These chromatin samples were then subjected to immunoprecipitation using a ChIP-grade H3 antibody and an appropriate IgG control. The relative amounts of viral DNA compared with cellular DNA in the immunoprecipitated material were assessed by qPCR using primers specific for the viral ORF47 gene and the cellular β2-microglobulin (B2m) gene, respectively (Fig. 4C). Background values derived from IgG controls were subtracted from values derived from H3 ChIP samples, and the data were subsequently normalized to cellular DNA by dividing the values for viral DNA by the values for cellular DNA. Since the doxycycline-treated samples contain newly amplified viral DNA, if this replicated DNA is associated with histone H3 then the relative amount of viral DNA immunoprecipitated with a H3 antibody in relation to cellular DNA should increase under these conditions, similar to the case for Fig. 4B. In fact, following immunoprecipitation, the relative amount of viral DNA detected decreased moderately at 24 and 48 h after viral reactivation, indicating that newly synthesized viral genomes in TREx BCBL-1-RTA cells are not associated with histone H3. Because the viral primers used in this analysis are specific to only one part of the viral genome, we also repeated the qPCR analysis using primers specific to the left viral origin of lytic replication (OriLytL), which is approximately 45 kb away from ORF47 in the viral genome (Fig. 4C). qPCR analysis of immunoprecipitated viral DNA using these primers yielded a similar trend, with a decrease in the relative amounts of viral DNA at 24 and 48 h following doxycycline treatment. Overall, these results indicate that KSHV evades deposition of cellular histones during lytic amplification of viral DNA.

### Functional ATM kinase and ATM-CHK2 activation is not essential for lytic replication of KSHV.

**FIG 5:**
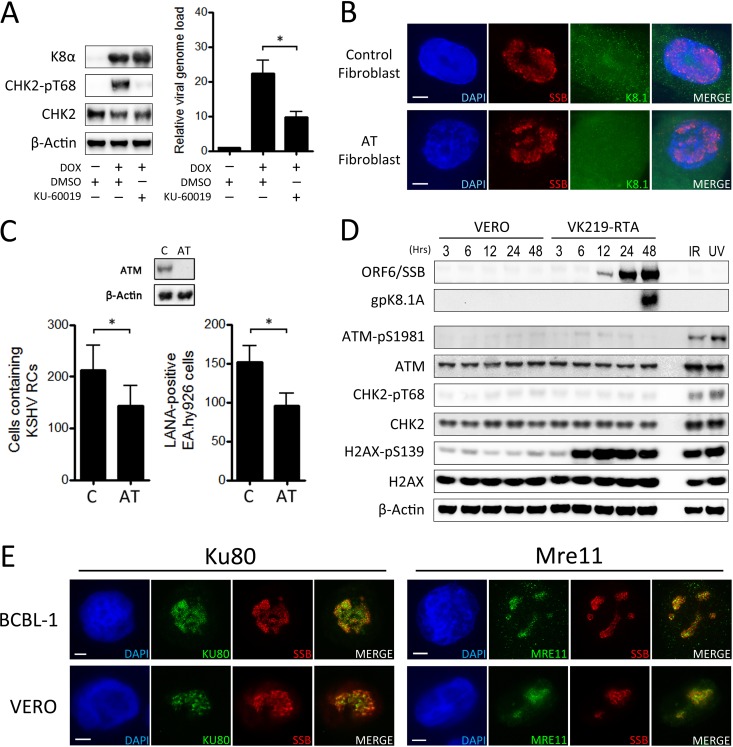
Contribution of the ATM kinase to efficient KSHV replication. (A) TREx BCBL-1-RTA cells were treated with either DMSO or 5 μM KU-60019 ATM kinase inhibitor prior to induction of lytic replication. The relative viral genome load was then measured after 48 h by qPCR, while Western blots were used to monitor levels of total and phosphorylated CHK2. Expression of viral K-bZIP was used to confirm lytic replication of KSHV. (B and C) Control and AT fibroblasts were *de novo* infected with KSHV on glass coverslips and treated with sodium butyrate for 72 h. (B) IF analysis was then carried out for expression of SSB and K8.1 lytic proteins. (C) The numbers of control and AT fibroblasts containing viral RCs were then counted across a coverslip in five repeat experiments. To assess release of infectious virus, supernatants from each cell line were then used to infect EA.hy926 cells, and the number of infected cells was determined after 48 h by IF analysis of LANA expression. All data bars represent the mean from five independent experiments, while error bars signify the SEM. *, *P* < 0.05 (statistical analyses were performed using a two-tailed and unpaired Student *t* test) (D) Vero and VK219-RTA cells were treated with doxycycline and sodium butyrate for the indicated times, and Western blotting was used to assess the levels of total and phosphorylated ATM, CHK2, and H2AX. VK219-RTA cells treated with either IR or UV were included as positive controls for DDR activation, while blotting for the lytic proteins SSB and K8.1A confirmed viral reactivation in VK219-RTA cells. (E) TREx BCBL-1-RTA cells were treated with doxycycline, while Vero cells were *de novo* infected with KSHV and treated with sodium butyrate. After 48 h, IF analysis was carried out for nuclear localization of Ku80 and MRE11 in relation to viral RCs marked by the viral SSB protein. Scale bars represent 5 μm.

Recognition of a DSB by MRN typically results in activation of the ATM kinase, which subsequently phosphorylates CHK2. We have previously reported that lytic replication of KSHV in TREx BCBL-1-RTA cells leads to activation of the ATM-CHK2 pathway and that attenuation of ATM signaling, using the specific ATM kinase inhibitor KU-55933, has a negative effect on release of infectious virus in these cells (33). Because the highest concentration of KU-55933 used previously was inefficient at inhibiting CHK2 phosphorylation and had a negative effect on cell viability, the restrictive effect of inhibiting ATM on KSHV replication in B cells was confirmed here using KU-60019, an improved analogue of KU-55933 that has been reported to be 10 times more effective at inhibiting phosphorylation of ATM targets than its predecessor (51). Application of 5 μM KU-60019 to TREx BCBL-1-RTA cells prior to induction of lytic replication inhibited phosphorylation of the ATM substrate CHK2 and led to a significant reduction in viral genome load compared to that with a dimethyl sulfoxide (DMSO) control (Fig. 5A).

To further assess the importance of the ATM kinase for KSHV lytic replication, ataxia-telangiectasia (AT) fibroblasts, which have a mutated form of the ATM gene, and control fibroblasts with functional ATM were grown on glass coverslips and infected with KSHV derived from TREx BCBL-1-RTA cells. The cells were subsequently treated with sodium butyrate, a histone deacetylase inhibitor known to promote lytic replication in latently infected cells (52). After 72 h, both cell lines were examined for expression of KSHV lytic proteins using IF microscopy (Fig. 5B). In both control and AT fibroblasts, cells containing KSHV RCs marked by viral SSB and expressing the late viral K8.1 protein were observed, indicating that both lines can support amplification of viral DNA and late lytic gene expression. To compare the numbers of cells from each fibroblast line containing lytic KSHV, the numbers of control and AT fibroblasts containing viral RCs were counted across a coverslip for each repeat experiment (Fig. 5C). To assess infectious virus release, the supernatants from these cells were applied to uninfected EA.hy926 cells, and the number of these cells infected with KSHV across a coverslip was determined after 48 h by using IF analysis of LANA expression (Fig. 5C). Although both cell lines clearly support KSHV lytic replication and production of infectious progeny, replication levels were significantly lower in AT cells than in the control line, suggesting that lack of functional ATM does have a negative effect on KSHV replication efficiency. These findings indicate that despite lacking functional ATM kinase, AT fibroblasts can support amplification of viral DNA during the lytic cycle, although at reduced levels compared to a control line with functional ATM.

We were also interested to determine if activation of the ATM-CHK2 pathway observed in B cells was a consistent event across other cell types. We therefore examined activation of the DDR during lytic replication in VK219-RTA cells, which are modified Vero cells containing a recombinant GFP/red fluorescent protein (RFP)-expressing KSHV and a doxycycline-inducible ORF50/RTA gene (53) (Fig. 5D). Although not a representative model for *in vivo* KSHV infection, this cell line is useful here because, unlike for many other latently infected cell lines, treatment of VK219-RTA cells with a combination of doxycycline and sodium butyrate results in highly efficient induction of the lytic replication program, which facilitates measurement of viral replication levels and detection of phosphorylated DDR proteins. Lytic replication was therefore induced in VK219-RTA cells, and phosphorylation of H2AX, CHK2, and ATM was assessed in comparison to that in uninfected Vero cells treated with the same concentrations of doxycycline and sodium butyrate. Detection of the lytic proteins SSB and K8.1A was used to confirm viral reactivation, while VK219-RTA cells exposed to either 20 J m^−2^ UV radiation for 6 h or 6 Gy IR for 1 h were used as positive controls for DDR activation. Induction of lytic replication in this cell line led to robust phosphorylation of H2AX, indicating DNA damage, although surprisingly, no obvious increase in phosphorylation of ATM or its substrate CHK2 could be observed. This indicates that activation of the ATM-CHK2 pathway during KSHV lytic replication is a cell line-specific event.

Since ATM activation is facilitated by recruitment of the MRN complex to DSBs, this led us to ask whether the localization of DSB-sensing proteins to viral RCs was also a cell line-specific occurrence. The localization of MRE11 and Ku80 during lytic replication was therefore examined in both TREx BCBL-1-RTA cells and Vero cells (Fig. 5E). Vero cells *de novo* infected with TREx BCBL-1-RTA-derived KSHV were used here, since the GFP/RFP-expressing recombinant virus contained within VK219-RTA cells is not compatible with three-color IF microscopy. As with EA.hy926-RTA cells, MRE11 and Ku80 were consistently localized to viral RCs in these cell lines, indicating that recruitment of DSB-sensing proteins to viral genomes is a consistent event during KSHV replication across multiple cell types. In summary, these findings suggest that although the ATM kinase may enhance KSHV replication in B cells and fibroblasts, ATM is not absolutely required for lytic replication of KSHV. In addition, since the MRN complex is associated with viral RCs in Vero cells, but the ATM-CHK2 pathway is not activated during KSHV replication in this cell line, it also appears that recognition of viral genomes by DSB recognition proteins is uncoupled from downstream DDR signaling.

### Depletion of MRE11 and inhibition of its exonuclease activity have a negative effect on viral replication.

**FIG 6:**
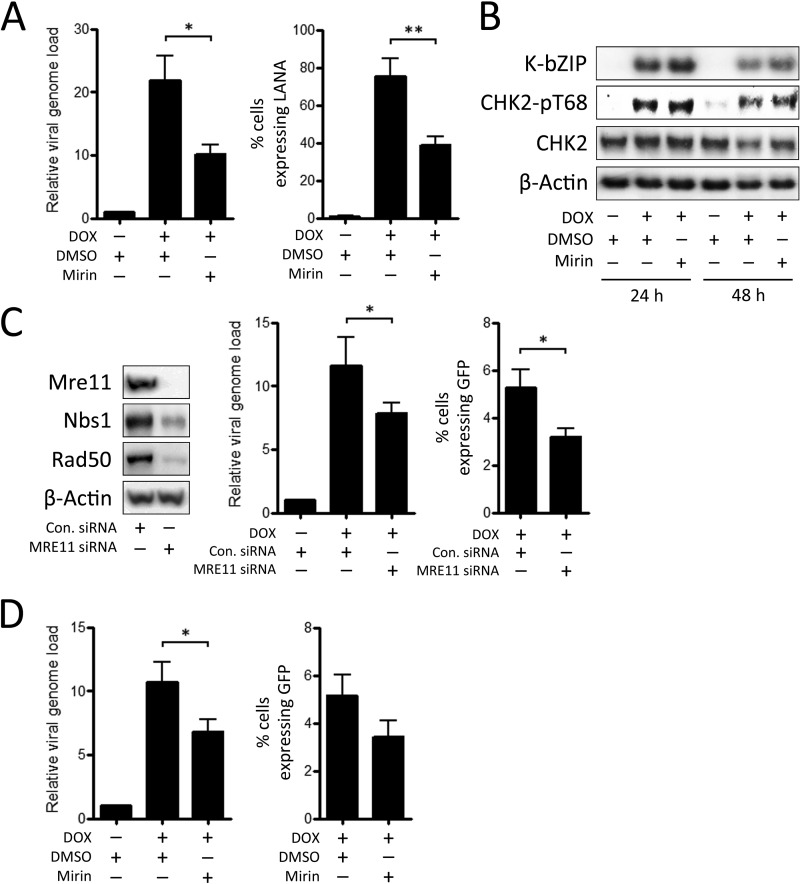
Effect of depletion of MRE11 and inhibition of its exonuclease activity on viral replication. (A and B) TREx BCBL-1-RTA cells were treated with either DMSO or 20 μM mirin prior to induction of lytic replication. (A) The relative viral genome load was then measured after 48 h by qPCR, while supernatants were then used to infect EA.hy926 cells, and the number of infected cells was determined after 48 h by IF analysis of LANA expression. (B) Western blots were used to assess levels of total and phosphorylated CHK2 at 24 and 48 h after doxycycline treatment. Expression of viral K-bZIP was used to confirm lytic replication of KSHV. (C) VK219-RTA cells were treated with siRNA against MRE11 or control siRNA for 72 h, and Western blotting was used to assess levels of MRN proteins. Following induction of lytic replication, the relative viral genome load was measured after 72 h by qPCR, while supernatants were then used to infect HEK293 cells, and the percentage of cells expressing GFP was determined after 48 h using flow cytometry. (D) VK219-RTA cells were treated with either DMSO or 20 μM mirin prior to induction of lytic replication. The relative viral genome load was measured after 72 h by qPCR, while supernatants were then used to infect HEK293 cells, and the percentage of cells expressing GFP was determined after 48 h using flow cytometry. All data bars represent the mean from three independent experiments, while error bars signify the SEM. *, *P* < 0.05; **, *P* < 0.01 (statistical analyses were performed using a two-tailed and unpaired Student *t* test).

Since the MRN complex and Ku70/80 are the only factors we have observed during this study that consistently colocalize with viral genomes, we were interested to determine how inhibition of these DDR proteins would affect KSHV replication. MRE11 has 3′-to-5′ exonuclease activity that is required for DNA resection prior to HR repair of DSBs (44). Due to the consistent localization of MRE11 to KSHV RCs, the effect of mirin, a specific inhibitor of MRE11 exonuclease activity, on viral replication was examined (Fig. 6A). Mirin was applied to TREx BCBL-1-RTA cells at a concentration of 20 μM at 1 h prior to induction of lytic replication using doxycycline. After 48 h, the relative viral genome load was measured by qPCR, while production of infectious virus was measured by applying infectious supernatants to EA.hy926 cells and calculating the percentage of cells expressing the latent viral protein LANA using IF microscopy. Application of mirin resulted in a consistent reduction in both viral genome amplification and production of infectious virus in TREx BCBL-1-RTA cells, indicating that the exonuclease activity of MRE11 is required for efficient viral replication.

Because the MRN complex facilitates recruitment and activation of ATM (14), we also examined whether mirin inhibits activation of the ATM-CHK2 pathway that occurs during KSHV replication in TREx BCBL-1-RTA cells. The effect of mirin on phosphorylation of CHK2 was therefore assessed in TREx BCBL-1-RTA cells at 24 and 48 h following doxycycline treatment (Fig. 6B). In contrast to the effect the ATM kinase inhibitor, there was no clear decrease in levels of phosphorylated CHK2 following mirin treatment at either time point examined. This is consistent with the previous finding that MRE11 exonuclease activity is dispensable for ATM activation (54) and indicates that the restrictive effect of mirin on viral replication in B cells is not due to impairment of ATM-CHK2 signaling during the lytic cycle.

Since inhibition of MRE11 exonuclease activity abrogated KSHV replication, the effect of small interfering RNA (siRNA) knockdown of MRE11 on viral replication was also assessed (Fig. 6C). Since TREx BCBL-1-RTA cells are relatively resistant to transfection and recruitment of MRE11 to viral RCs is consistent across different cell types, these experiments were performed in VK219-RTA cells. SmartPool siRNAs directed against MRE11 and control nontargeting siRNAs were applied to VK219-RTA cells for 72 h, and Western blotting was used to confirm depletion of MRE11. Knockdown of MRE11 is known to reduce stability of the other MRN components, NBS1 and RAD50 (55). As expected, siRNA knockdown of MRE11 in VK219-RTA cells also led to a reduction in levels of NBS1 and RAD50. Following MRE11 depletion, lytic replication was induced in these cells, and the relative viral genome load was then measured by qPCR after 72 h. Because this cell line contains a recombinant KSHV that expresses GFP during latency, production of infectious virus from these cells was measured by infecting HEK293 cells with infectious supernatants and determining the percentage expressing GFP 48 h later using flow cytometry. Depletion of MRE11 resulted in a significant reduction in both viral genome amplification and release of infectious virus in VK219-RTA cells, although this decrease was less pronounced than the effect of mirin in TREx BCBL-1-RTA cells.

The effect of mirin on viral replication in VK219-RTA cells was also examined (Fig. 6D). Mirin was applied 1 h prior to viral reactivation, and viral genome amplification and infectious virus release were measured by qPCR and flow cytometry, respectively. Similar to the case in TREx BCBL-1-RTA cells, mirin had a negative effect on viral genome amplification and release of infectious virus in VK219-RTA cells, although only the decrease in relative viral genome load reached statistical significance. Overall, these results indicate that the MRE11 protein contributes to efficient lytic replication of KSHV.

### Knockdown of Ku80 and inhibition of DNA-PK enhances viral replication.

**FIG 7:**
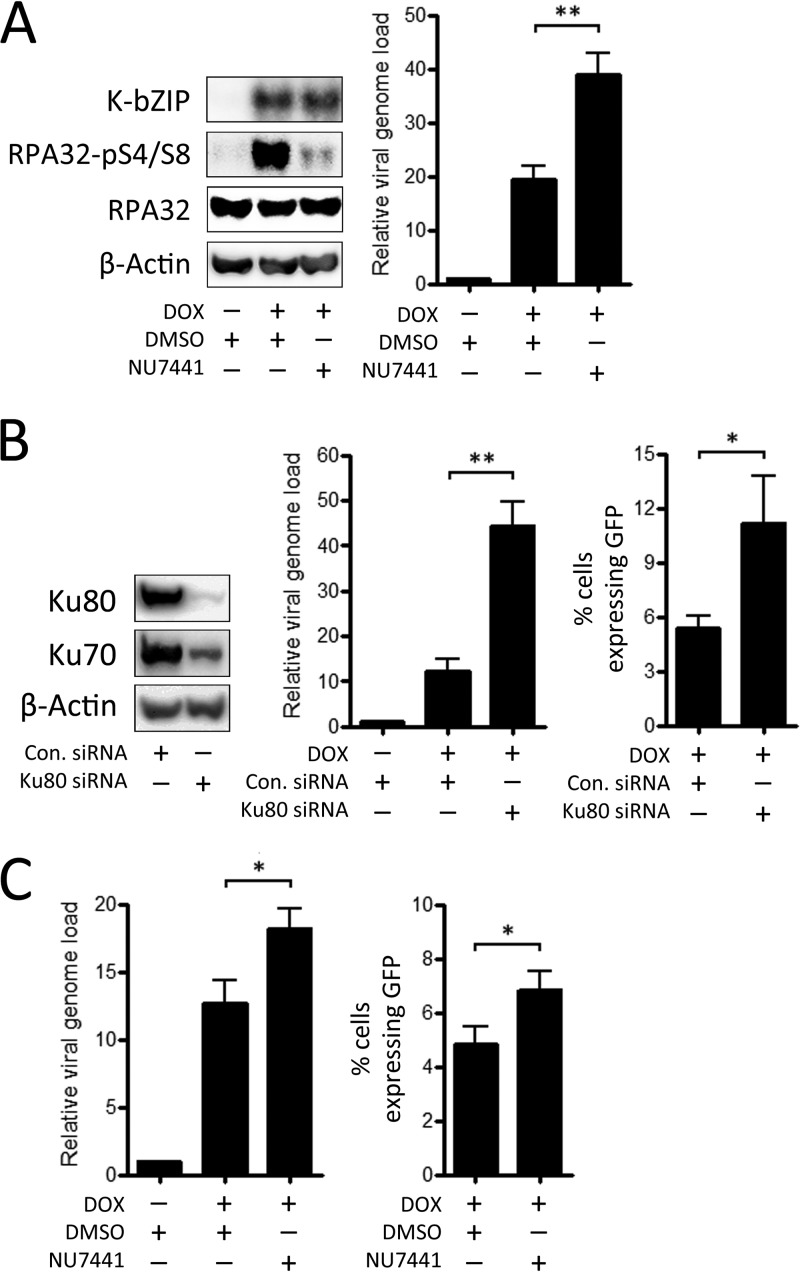
Effect of depletion of Ku80 and inhibition of DNA-PK activity on viral replication. (A) TREx BCBL-1-RTA cells were treated with either DMSO or 1 μM NU7441 DNA-PK inhibitor prior to induction of lytic replication. The relative viral genome load was then measured after 48 h by qPCR, while Western blots were used to monitor levels of total and phosphorylated RPA32. Expression of viral K-bZIP was used to confirm lytic replication of KSHV. (B) VK219-RTA cells were treated with siRNA against Ku80 or control siRNA, and Western blotting was used to monitor levels of Ku80 and Ku70 after 72 h. Following induction of lytic replication, the relative viral genome load was measured after 72 h by qPCR, while supernatants were then used to infect HEK293 cells, and the percentage of cells expressing GFP was determined after 48 h using flow cytometry. (C) VK219-RTA cells were treated with either DMSO or 1 μM NU7441 DNA-PK inhibitor prior to induction of lytic replication. After 72 h, qPCR was used to assess the relative genome load, while supernatants were then used to infect HEK293 cells, and the percentage of cells expressing GFP was determined after 48 h using flow cytometry. All data bars represent the mean from three independent experiments, while error bars signify the SEM. *, *P* < 0.05; **, *P* < 0.01 (statistical analyses were performed using a two-tailed and unpaired Student *t* test).

Our previous findings demonstrated that inhibition of DNA-PK in TREx BCBL-1-RTA cells using the specific inhibitor NU7441 resulted in increased release of infectious virus and enhanced phosphorylation of ATM substrates during lytic replication of KSHV (33). To confirm that DNA-PK inhibition can enhance amplification of KSHV DNA in these cells, the effect of NU7441 on the relative viral genome load was assessed by qPCR (Fig. 7A). Addition of NU7441 to TREx BCBL-1-RTA cells prior to doxycycline treatment resulted in a dramatic reduction in the phosphorylation of RPA32 at serines 4 and 8 that occurs during viral replication in these cells, confirming that this phosphorylation event is attributable to DNA-PK kinase activity. NU7441 also led to an increase in relative viral genome load at 48 h following addition of doxycycline compared with the DMSO control, indicating that DNA-PK activity has a negative effect on KSHV replication.

After binding to DNA ends, the Ku70/80 heterodimer recruits DNA-PKcs to form the DNA-PK enzyme, which participates in NHEJ. Due to its consistent localization to KSHV RCs and its association with DNA-PKcs, the effect of Ku80 knockdown on KSHV lytic replication was also assessed (Fig. 7B). As with depletion of MRE11, Ku80 knockdown was carried out in VK219-RTA cells at 72 h prior to induction of lytic replication, and Western blotting was used to monitor levels of Ku80 and its binding partner, Ku70. Since the interaction between Ku70 and Ku80 promotes stability of both proteins (56), depletion of Ku80 also led to a reduction in levels of Ku70 in VK219-RTA cells. As before, lytic replication was then induced in these cells, and the relative genome load and release of infectious virus were assessed by qPCR and flow cytometry, respectively, after 72 h. In contrast to the negative effect of MRE11 knockdown, reduction of Ku80 led to a dramatic increase in the viral genome load after 72 h. Ku80 depletion also led to increased release of infectious virus from these cells, although the relative increase observed was less than the increase in viral genome load.

The effect of DNA-PK kinase inhibition on viral replication in VK219-RTA cells was also evaluated by application of NU7441 prior to induction of viral replication (Fig. 7C). As with TREx BCBL-1-RTA cells, application of this compound to VK219-RTA cells resulted in a significant increase in viral genome load and release of infectious virus at 72 h after lytic reactivation. Overall, these findings indicate that, in contrast to the case for ATM and MRE11, components of the NHEJ pathway exert a restrictive effect on KSHV replication.

## DISCUSSION

Here we have presented a comprehensive assessment of the localization of DSB repair factors during lytic replication of KSHV. We have confirmed that the complete MRN complex and the Ku70/80 heterodimer, which recognize and bind DSBs, consistently colocalize with viral genomes as RCs expand to fill the nuclear space. Interestingly, BRCA1 and CtIP, which can form a complex with MRN during DNA repair, were also observed at KSHV RCs but not as consistently as the MRN complex. The interaction between these proteins and MRN relies on the phosphorylation of CtIP by CDKs, which restricts formation of the complex, and therefore DNA resection, to the S and G_2_ phases of the cell cycle (57). Previous research has indicated that lytic replication of KSHV occurs in G_1_, which would explain the lack of localization of these factors to RCs (58). However, our earlier findings indicate that in B cells, induction of lytic replication leads to an increase in the number of cells in S phase (33). It is possible, therefore, that KSHV lytic replication can occur in both G_1_ and S phases and that localization of BRCA1 and CtIP to viral RCs is cell cycle dependent. Whether recruitment of CtIP and BRCA1 to RCs has a positive or negative effect on viral genome replication was not evaluated here but would be an interesting avenue of investigation for future research.

With the exception of RAD52, other factors examined that participate in HR repair downstream of DNA end resection did not localize to viral RCs. In the case of RAD51, the lack of recruitment to viral genomes was unexpected considering that this protein has been observed at sites of both HSV-1 and EBV DNA synthesis and has also been implicated in efficient replication of both of these herpesviruses (38, 39, 59). In the case of HSV-1, knockdown of the HR factors RAD51 and RAD52 abrogates recombination events between viral genomes, while a separate study demonstrated that individual depletion of RAD51, RAD52, and RAD54 dramatically reduced HSV-1 replication levels following exposure of the viral genomes to UV radiation (46, 59). While these studies have implicated cellular HR factors in viral replication, it has also been shown that the HSV-1 ssDNA binding protein ICP8 and the viral alkaline nuclease UL12 can form a two-component recombinase that catalyzes *in vitro* strand exchange reactions similarly to the phage lambda Red α/β (60). UL12 and ICP8 also interact with members of the MRN complex, raising the possibility that viral and cellular proteins can cooperate to promote recombination events that enhance the fidelity of herpesvirus replication (61, 62).

The formation of γH2AX, MDC1, and 53BP1 foci outside viral RCs suggests that cellular DNA damage can occur during the KSHV lytic replication phase. The occurrence of DNA damage during KSHV replication has previously been demonstrated using comet assays, while the lytic ORF57/MTA protein has also been shown to cause DNA damage when ectopically expressed in HEK293 cells (41, 63). Confocal images from the present study confirm that DSB repair factors aggregate on the margins of viral RCs, although it is not currently clear why DNA damage would be concentrated in these areas. One explanation is that the rapid expansion of these viral replication structures causes physical compaction of cellular chromatin resulting in DNA fragmentation. Despite this, it is unlikely that expansion of RCs would be the sole cause of cellular DNA damage in this setting. This is because we have previously demonstrated that inhibition of viral DNA amplification, and therefore expansion of viral RCs, in B cells does not reduce phosphorylation of DDR proteins following lytic reactivation (33). Based on this observation, as well as the previous identification of herpesvirus proteins that can cause DNA damage when expressed alone, it is possible that numerous events unique to the lytic replication phase can contribute to the formation of cellular DNA lesions.

We have also presented evidence here that newly amplified KSHV DNA is not associated with cellular histones. Histone deposition on replicating KSHV genomes has not previously been examined, although the fact that KSHV DNA has been shown to be histone free in the virion particle provides additional evidence of histone exclusion during the lytic cycle (64, 65). Posttranslational modifications of histones that flank sites of DSBs play critical roles in regulating assembly of repair complexes and in orchestrating cell cycle checkpoint activation (66). The lack of histones on viral DNA would explain why proteins such as MDC1 and 53BP1 are not concentrated at viral RCs despite the apparent occurrence of DSBs in viral DNA that are recognized by the DDR, since these proteins are recruited to DSBs following recognition of histones modifications at break sites (18, 19). Although localization of ATM was not examined here, it is possible that this kinase is recruited to viral DSBs even though viral replication does not result in stimulation of ATM-CHK2 signaling. This is because, while MRN can recruit ATM to DSBs together with the TIP60 acetyltransferase, efficient activation of ATM kinase activity requires an interaction between TIP60 and histone H3 trimethylated on lysine 9 (H3K9me3) (67). Formation of phosphorylated ATM foci has also been shown to be dependent on the interaction between H2AX and MDC1 that occurs following DSBs (18). Ultimately, the absence of histone deposition by KSHV may be part of a strategy to avoid localization of DNA repair factors that would otherwise lead to detrimental processing of viral DNA.

Ambiguity regarding the requirement of the ATM kinase for lytic replication of herpesviruses is a recurring theme in studies regarding these pathogens and the DDR. We have shown here, and in more depth previously, that lytic reactivation in B cells leads to robust activation of the ATM-CHK2 pathway and that inhibition of this response has a negative effect on viral replication in this cell line. Results derived using AT fibroblasts, which lack functional ATM, demonstrate that although ATM may enhance KSHV replication, it is not absolutely required for completion of the lytic cycle. The further finding that ATM-CHK2 activation is a cell line-specific event also highlights the redundancy of this signaling pathway for amplification of viral DNA and release of infectious progeny. It is currently unclear why ATM is activated during KSHV replication in B cells but not in Vero cells despite robust phosphorylation of H2AX in both cell lines indicating DNA damage. However, the consistent recruitment of DSB-sensing proteins to viral RCs in Vero cells in the absence of ATM-CHK2 signaling does demonstrate that recognition of viral DSBs by the cellular DDR is uncoupled from activation of this downstream signaling pathway.

We have also assessed the contribution of DSB repair factors to efficient replication of KSHV. This assessment was restricted to those proteins that consistently localized to viral RCs as well as proteins for which a specific inhibitor was commercially available. While application of the MRE11 exonuclease inhibitor reduced levels of viral replication in B cells by approximately 50%, knockdown of MRE11 in VK219 cells had a more moderate negative effect despite efficient depletion of this protein prior to viral reactivation. It is possible that only small amounts of MRE11 are necessary for viral replication, although since MRE11-null cells are not viable, it is difficult to ascertain how complete loss of this protein would impact synthesis of viral DNA. Although localization of MRN to viral RCs has been observed in several species of herpesvirus (35, 38), a specific role for this complex during lytic replication of these pathogens has yet to be identified. Interestingly, MRN proteins have antiviral functions at an earlier stage in the herpesvirus life cycle, as it has been demonstrated that a RAD50-MRE11-CARD9 complex can recognize KSHV DNA in the cytoplasm and trigger activation of the NF-κB pathway to suppress viral replication (68, 69). MRN can also abrogate replication of adenovirus DNA, and it has recently been shown that an interaction between MRN and adenovirus genomes promotes a limited ATM response that specifically restricts viral but not cellular DNA replication (70). Serotype-specific degradation, redistribution, and inactivation of MRN components during adenovirus infection have also previously been documented (71–73). While MRN has a well-defined role in DSB detection and repair, the complex has also been shown to associate with replicating DNA during S phase and to promote replisome stability and replication restart following fork stalling (74, 75). It is conceivable that MRN contributes similarly to replication of herpesvirus DNA, although why this cellular complex is apparently beneficial to some double-stranded DNA viruses while detrimental to others remains enigmatic.

Depletion of the NHEJ factor Ku80 resulted in an increase in viral DNA amplification and release of infectious virus in VK219-RTA cells, while inhibition of DNA-PK enzymatic activity also enhanced viral replication in both VK219-RTA and TREx BCBL-1-RTA cells. DNA affinity purification and mass spectrometry have previously been used to show that both the Ku70/80 heterodimer and DNA-PKcs bind to the KSHV origin of lytic replication in induced BCBL-1 cells, indicating that the complete DNA-PK holoenzyme can interact with KSHV genomes following lytic reactivation (76). Further evidence for a restrictive role for DNA-PK in KSHV replication comes from a previous study showing that the viral PF8 protein blocks the interaction between Ku70/80 and DNA-PKcs, resulting in inhibition of NHEJ (63). The findings presented here are also in agreement with research in HSV-1 demonstrating that viral replication is enhanced in Ku-deficient murine embryonic fibroblasts and in DNA-PKcs-deficient glioma cells (62, 77). None of these previous investigations have clarified exactly how the NHEJ pathway attenuates replication of herpesvirus DNA, although it has been suggested that activation of this pathway could lead to inappropriate ligation of viral replication products during cleavage of concatemeric DNA (77, 78).

**FIG 8:**
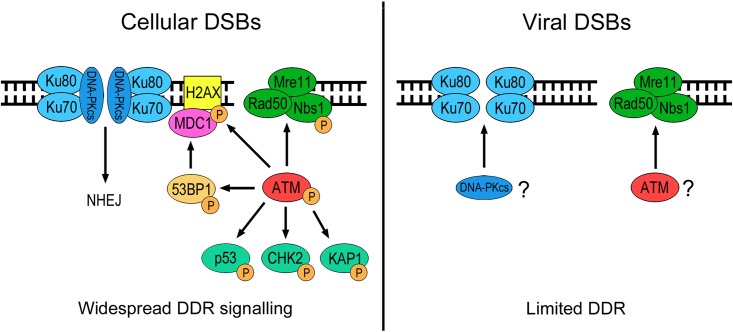
Model of the DDR to cellular and viral DSBs. Cellular DSBs elicit ATM and DNA-PK activation leading to widespread DDR signaling. In contrast, DSBs in viral DNA result in a more limited DDR involving recruitment of DSB-sensing complexes but not downstream phosphorylation of transducer and effector proteins.

In summary, the results presented in this study indicate that DSBs form in viral DNA during lytic replication of KSHV and elicit a host DDR that is distinct from the response to cellular DSBs (Fig. 8). Cellular DSBs typically result in activation of ATM and DNA-PK, leading to phosphorylation of multiple substrates that orchestrate assembly of DNA repair foci and cell cycle checkpoint activation. In contrast, viral DNA breaks result in a more limited DDR that involves localization of sensor proteins, potentially DNA-PK and ATM, but not downstream phosphorylation of transducer and effector proteins.

It is now established that DSB repair proteins localize to KSHV genomes in the cytoplasm and cooperate in cellular antiviral immune responses (31, 68). The findings presented here indicate that the DDR also exerts an antiviral effect during lytic replication through components of the NHEJ repair pathway. While we have not demonstrated conclusively that specific DDR factors are essential for KSHV lytic replication, it appears that ATM and MRE11 promote efficient synthesis of viral genomes and release of infectious progeny. Overall, this study highlights the complex relationship between KSHV and the cellular DSB repair machinery and further demonstrates how lytic replication of herpesviruses inevitably involves selective interactions with host DDR signaling pathways.

## MATERIALS AND METHODS

### Cell culture and drug additions.

All cell lines used in this study were cultured in a humidified incubator at 37°C with 5% CO_2_. TREx BCBL-1-RTA cells (developed by Jae Jung, University of Southern California, USA) (79) were maintained in RPMI growth medium (Sigma-Aldrich) supplemented with 10% fetal bovine serum (FBS) (Sigma-Aldrich), 1% penicillin-streptomycin (Gibco), and 100 μg/ml of hygromycin B (Roche). EA.hy926 cells (purchased from ATCC), HEK293 cells (purchased from ATCC), and ataxia-telangiectasia (AT) and control dermal fibroblasts (provided by Malcolm Taylor, University of Birmingham, UK) were cultured in Dulbecco modified Eagle medium (DMEM) (Sigma-Aldrich) supplemented with 10% FBS and 1% penicillin-streptomycin. EA.hy926-RTA cells, which express ORF50/RTA under the control of the tetracycline promoter, were also cultured in the presence of 250 μg/ml of G418 (Sigma-Aldrich). VK219-RTA cells (provided by Andrew Hislop, University of Birmingham, UK), which contain the recombinant rKSHV.219 virus and express ORF50/RTA under the control of the tetracycline promoter, were maintained in AQ medium (Sigma-Aldrich) supplemented with 10% FBS, 1% penicillin-streptomycin, 5 μg/ml puromycin (Sigma-Aldrich), and 250 μg/ml G418. Vero cells were cultured in the same medium but in the absence of G418 and puromycin. U2OS cells expressing doxycycline-inducible GFP-tagged CtIP (developed by Alessandro Sartori, University of Zurich, Switzerland) (45) were cultured in DMEM supplemented with 10% FBS, 1% penicillin-streptomycin, 100 μg/ml hygromycin B, and 5 μg/ml blasticidin (Sigma-Aldrich). Latent KSHV within TREx BCBL-1-RTA and EA.hy926-RTA cells was reactivated by the addition of 0.5 μg/ml doxycycline, while VK219-RTA cells were induced by the addition of 1 μg/ml doxycycline and 0.5 mM sodium butyrate.

Stock solutions of the MRE11 exonuclease inhibitor mirin, the ATM kinase inhibitor KU-60019, and the DNA-PK inhibitor NU7441 (all purchased from Tocris Bioscience) were prepared in DMSO. These compounds, and appropriate DMSO vehicle controls, were added to cells 1 h prior to stimulation of lytic replication. Supernatants were collected at the indicated time points and assessed for infectious virus levels, while cells were also harvested and evaluated for relative viral genome load using qPCR as outlined below.

### *De novo* infection of cells with KSHV.

In order to quantify infectious virus production from induced VK219-RTA cells, supernatants containing infectious KSHV were added directly to HEK293 cells in culture plates that were then spinoculated (330 × *g*, 20 min, room temperature) and cultured for 4 h at 37°C. Virus-containing supernatants were then removed and replaced with normal supplemented medium. After 48 h, cells were harvested and fixed in 4% paraformaldehyde, and a minimum of 10,000 cells were assessed to determine the percent GFP expression relative to that in uninfected controls using an Accuri C6 flow cytometer (BD). Quantification of infectious virus production from induced TREx BCBL-1-RTA cells was carried out as described above except that supernatants were added to EA.hy926 cells which were subsequently analyzed for expression of the viral protein LANA using the IF microscopy protocol outlined below. In this case, a minimum of 500 cells were analyzed in each of three independent experiments.

To assess KSHV replication in fibroblasts, TREx BCBL-1-RTA cells were resuspended in antibiotic-free medium containing 0.5 μg/ml doxycycline at a density of 5 × 10^5^ cells/ml. After 72 h, cells were pelleted and supernatant containing infectious KSHV was applied directly to AT and control fibroblasts grown on 13 mm coverslips in 24-well plates. Cells were spinoculated (330 × *g*, 20 min, room temperature) and cultured for 6 h at 37°C. After 24 h, infectious supernatants were removed, cells were washed twice with phosphate-buffered saline (PBS), and fresh culture medium containing 0.5 mM sodium butyrate (Sigma-Aldrich) was added. After a further 72 h, cells were fixed for assessment of viral gene expression using IF microscopy. To assess production of infectious virus from these cells, supernatants were added directly to EA.hy926 cells in culture plates which were spinoculated as outlined above. After 48 h, EA.hy926 cells were then analyzed for LANA expression using the IF microscopy protocol outlined below.

In order to visualize KSHV RCs, EA.hy926-RTA cells were grown on glass coverslips and infected with TREx BCBL-1-RTA-derived KSHV as outlined above except that cells were cultured with the virus for 4 h at 37°C before infectious supernatants were removed and replaced with fresh culture medium. After an additional 24 h, cells were treated with 0.5 μg/ml doxycycline for 48 h to induce lytic replication before being fixed and stained as outlined below.

### IF microscopy.

Adherent cells were grown on glass coverslips in 24-well plates, while suspension cells were cytospun onto glass slides. Cells were either fixed in 4% paraformaldehyde in PBS for 10 min and permeabilized in 0.5% Triton X-100 for 5 min in PBS or preextracted on ice for 5 min with nuclear extraction buffer [10 mM piperazine-*N*,*N*′-bis(2-ethanesulfonic acid) (PIPES), 20 mM NaCl, 3 mM MgCl_2_, 300 mM sucrose, 0.5% Triton X-100] before being fixed in 4% paraformaldehyde in PBS for 10 min. Cells were blocked in 10% heat-inactivated goat serum (HINGS) for 30 min before addition of primary antibodies diluted in 10% HINGS for 1 h at room temperature. Cells were washed three times in PBS before addition of Alexa Fluor-conjugated secondary antibodies diluted in 10% HINGS for 1 h at room temperature. Cells were washed three times in PBS before addition of DAPI (4′,6′-diamidino-2-phenylindole) nuclear stain diluted in PBS for 1 min (Life Technologies). Coverslips were mounted using Prolong Gold antifade reagent (Life Technologies). Fluorescence images were taken using a Leica DM6000B epifluorescence microscope with Leica Application Suite Advanced Fluorescence software. For confocal immunofluorescence microscopy (IF), EA.hy926-RTA cells were prepared as described above. z-stack images made up of 1-μm optical sections were then generated using a Nikon A1R confocal laser microscope system with Nikon NIS-Elements software and processed using ImageJ software.

The following primary antibodies were used for IF microscopy: anti-LANA (NCL-HHV8-LNA; Novacastra), anti-ORF6/SSB (provided by Gary Hayward, Johns Hopkins University, MD, USA), anti-ORF59/PF8 (in-house), anti-K8.1 (in-house), anti-MRE11 (GTX70212; Genetex), anti-NBS1 (GTX70222; Genetex), anti-RAD50 (ab89; Abcam), anti-Ku80 (GTX70276; Genetex), anti-Ku70 (ab83501; Abcam), anti-BRCA1 (sc-6954; Santa Cruz), anti-RAD51 (PC130; Merck Millipore), anti-RAD52 (sc-8350; Santa Cruz), anti-RAD54B (GTX103291; Genetex), anti-γH2AX (S139) (05-636; Merck Millipore), anti-MDC1 (in-house), anti-53BP1 (ab36823; Abcam), anti-histone H1 (39708; Active Motif), and anti-histone H3 (GTX122148; Genetex).

### siRNA transfections.

siRNA On-Target Plus Smart pools targeting XRCC5 (Ku80) (L-010491-00-0005) and MRE11A (L-009271-00-0005), as well as a nontargeting control pool (D-001810-10-05) (Dharmacon), were resuspended to 20 μM stock solutions in siRNA buffer (Dharmacon). VK219-RTA cells were transfected with siRNAs at a concentration of 10 nM in 6-cm dishes using Lipofectamine RNAiMAX (Invitrogen) according to the manufacturer's instructions. siRNAs and RNAiMAX were diluted in Opti-MEM (Gibco) and applied directly to cell culture medium following 20 min of incubation at room temperature. After 72 h, the medium was replaced, lytic reactivation was induced by the addition of 1 μg/ml doxycycline and 0.5 μM sodium butyrate, and cells were left for an additional 72 h. Supernatants were then collected for analysis of infectious virus production using flow cytometry, while cells were harvested for analysis of relative viral genome load by qPCR.

### Western blot analysis.

Cell lysates were prepared by sonicating in UTB buffer (8 M urea, 50 mM Tris HCl [pH 7.4], 150 mM β-mercaptoethanol). Following protein concentration determination using the Bradford reagent (Bio-Rad), lysates were suspended in 2× Laemmli sample buffer (Bio-Rad) and heated to 95°C for 10 min. Lysates were then subjected to sodium dodecyl sulfate-polyacrylamide gel electrophoresis (SDS-PAGE) using standard procedures.

The following primary antibodies were used for Western blotting: anti-K8α/K-bZIP (SAB5300152; Sigma-Aldrich), anti-ORF6/SSB (provided by Gary Hayward), anti-K8.1A (in-house), anti-β-actin (A2228; Sigma-Aldrich), anti-ATM (2873; Cell Signaling), anti-phospho-ATM (S1981) (AF1655; R&D Systems), anti-CHK2 (2662; Cell Signaling), anti-phospho-CHK2 (T68) (2661; Cell Signaling), anti-RPA32 (NA19L; Calbiochem), anti-phospho-RPA32 (S4/S8) (A300-245A; Bethyl), anti-H2AX (7631; Cell Signaling), anti-γH2AX (S139) (05-636; Merck Millipore), anti-MRE11 (GTX70212; Genetex), anti-NBS1 (GTX70222; Genetex), anti-RAD50 and anti-KU80 (sc-6954; Santa Cruz), and anti-KU70 (ab83501; Abcam). Goat anti-mouse and swine anti-rabbit horseradish peroxidase (HRP)-conjugated secondary antibodies (Dako Laboratories) and ECL detection reagent (GE Healthcare) were used to visualize proteins on a Fusion SL chemiluminescence imaging system (Vilber Lourmat).

### qPCR.

To assess the relative viral genome load in KSHV-infected cells, DNA was extracted using a Wizard genomic DNA purification kit (Promega) according to the manufacturer's instructions and quantified by UV spectrophotometry. Amplification reactions were performed in 20-μl volumes containing 10 μl SensiFAST SYBR master mix (Bioline), 0.1 μM forward and reverse primers, and 50 ng DNA template. Cycling was performed on an Applied Biosystems 7500 Fast Dx real-time PCR machine using an initial denaturation at 94°C for 3 min followed by 40 cycles of a 5-s denaturation at 94°C, a 10-s annealing at 60°C, and a 20-s extension at 72°C. Samples were run in triplicate, and dissociation curve analysis was used to confirm that each primer pair generated a single product. The viral genome load was then calculated relative to that in uninduced cells containing latent KSHV. Viral DNA was detected using primers specific to the KSHV ORF47 gene (sequences provided by Adrian Whitehouse, University of Leeds, UK) and normalized to cellular DNA using previously published primers to the cellular β2-microglobulin (B2m) gene (80). Fold changes relative to uninduced controls were then calculated using the comparative threshold cycle (*C_T_*) method. For ChIP experiments, viral DNA was also detected using previously published primers against the KSHV left origin of lytic replication (OriLytL) (5). Primers used in quantitative PCRs (qPCRs) were B2m-Fwd (5′-GGAATTGATTTGGGAGAGCATC3′) and B2m-Rev (5′-CAGGTCCTGGCTCTACAATTTACTAA-3′), ORF47-Fwd (5′-CGCGGTCGTTCGAAGATTGGG-3′) and ORF47-Rev (5′-CGAGTCTGACTTCCGCTAACA-3′), and OriLytL-Fwd (5′-GGGGGGGGGCTAGTGAGTCA-3′) and OriLytL-Rev (5′-GTAACAGTTGGTTAACCCGT-3′).

### ChIP.

TREx BCBL-1-RTA cells were treated with 0.5 μg/ml doxycycline for 0, 24, and 48 h. Chromatin immunoprecipitation (ChIP) was then carried out using an Active Motif Chip-It express kit according to the manufacturer's instructions. In brief, 1.5 × 10^7^ cells per condition were cross-linked for 10 min in 1% formaldehyde before neutralization with 0.125 M glycine. Following lysis, cells were subjected to sonication for 8 min (30 s on and 30 s off). A proportion of the chromatin from each condition was cleaned up using phenol-chloroform extraction to provide input DNA samples that were subsequently subjected to qPCR analysis of relative viral genome load as outlined above. Chromatin-protein complexes were immunoprecipitated from the remaining chromatin using protein G-coated magnetic beads (Active Motif) and antibodies against histone H3 (D2B12; Cell Signaling) or normal rabbit IgG (2729; Cell Signaling). Following washing and elution of the protein-DNA complexes, cross-links were reversed by heating and treatment with proteinase K (Active Motif).

The relative quantities of viral and cellular DNA fragments present following immunoprecipitations were determined using qPCRs as outlined above. Background values obtained from immunoprecipitation reactions using the control IgG antibody were subtracted from the values obtained using the antibody against histone H3. Values for viral DNA at each time point were also normalized to values derived from cellular DNA to account for differences in immunoprecipitation efficiencies.
